# Supplementary material for: Metal 3D nanoprinting with coupled fields
Source: Nat Commun. 2023 Aug 15;14:4920. doi: 10.1038/s41467-023-40577-3 (PMC10427678; doi:10.1038/s41467-023-40577-3)
Supplement: Supplementary file 1 — Supplementary Information [file 41467_2023_40577_MOESM1_ESM.pdf]

Supplementary Information for

Metal 3D nanoprinting with coupled fields

Bingyan Liu[1], Shirong Liu[1], Vasanthan Devaraj[2], Yuxiang Yin[1], Yueqi Zhang[1], Jingui Ai[1], Yaochen Han[1], Jicheng Feng[1]*

[1]School of Physical Science and Technology, ShanghaiTech University, Shanghai, China

[2] Bio-IT Fusion Technology Research Institute, Pusan National University, Busan, Republic of Korea

*Corresponding author. email: fengjch@shanghaitech.edu.cn

The main manuscript associated with this file can be found at https://doi.org/10.1038/s41467-023-40577-3

Table of Contents

Supplementary Figures S4
Supplementary Fig. 1: Schematic of our homemade 3D nanoprinter for coupling the electric and flow fields while exerting the key ability for size selection of NPs and their in-situ printing. S4
Supplementary Fig. 2: Size distribution of NPs measured by the NANO-SMPS and via analyzing their TEM (after size selection) images. S5
Supplementary Fig. 3: The effect of introducing a sheath flow used in tandem with electric fields. S6
Supplementary Fig. 4: Schematic of printing zone. S7
Supplementary Fig. 5: Interior nanostructures after FIB milling. S8
Supplementary Fig. 6: Arrayed nanowires with different bending angles and intergaps. S9
Supplementary Fig. 7: Nanoscale precision. S10
Supplementary Fig. 8: Multimaterials printing. S11
Supplementary Fig. 9: Printing metal 3D nanostructures over large areas. S12
Supplementary Fig. 10: Marked sizes for evaluating the cushion field. S15
Supplementary Fig. 11: 3D nanoprinting of different metals. S16
Supplementary Fig. 12: EDS analysis of metal 3D nanostructures. S17
Supplementary Fig. 13: IR measurements of the metal nanostructures S18
Supplementary Fig. 14: Simulated results for light interactions with the metal 3D nanostructures. S19
Supplementary Fig. 15: REELS measurements for the Pd nanostructures. S20
Supplementary Fig. 16: Protocols for surface protection. S21
Supplementary Tables S22
Supplementary Table 1 Tilt angles used in SEM imaging S22
Supplementary Table 2 Pattern design S23
Supplementary Table 3 Experimental parameters for printing multimaterials nanostructures S24
Supplementary Table 4 Flow rates and potentials used in printing S26
Supplementary Table 5 Pattern designs for the substrates used for printing the structures shown in Figs. 3 and 4. ..... S27
Supplementary Discussion S29
Supplementary Discussion 1. Particle size-selection with coupled flow and electric fields S29
Supplementary Discussion 2. Methodology of printing uniform nanostructures over large areas S30
Supplementary References S30

Supplementary Figures

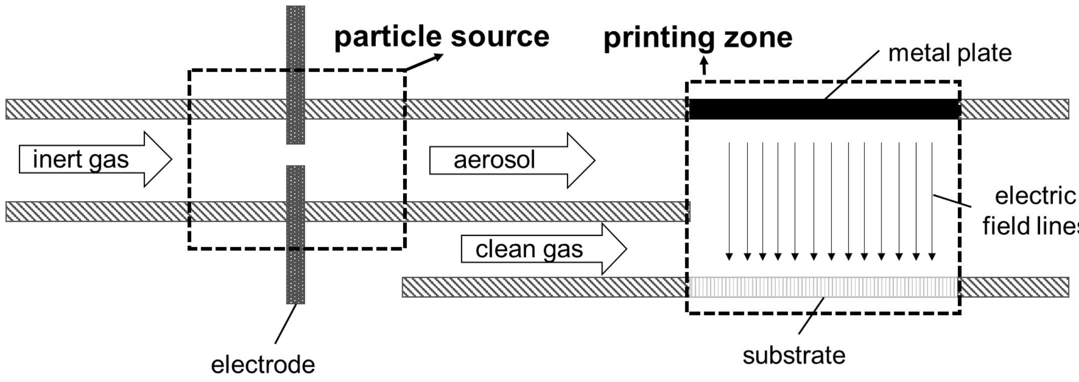

Supplementary Fig. 1: Schematic of our homemade 3D nanoprinter for coupling the electric and flow fields while exerting the key ability for size selection of NPs and their in-situ printing.

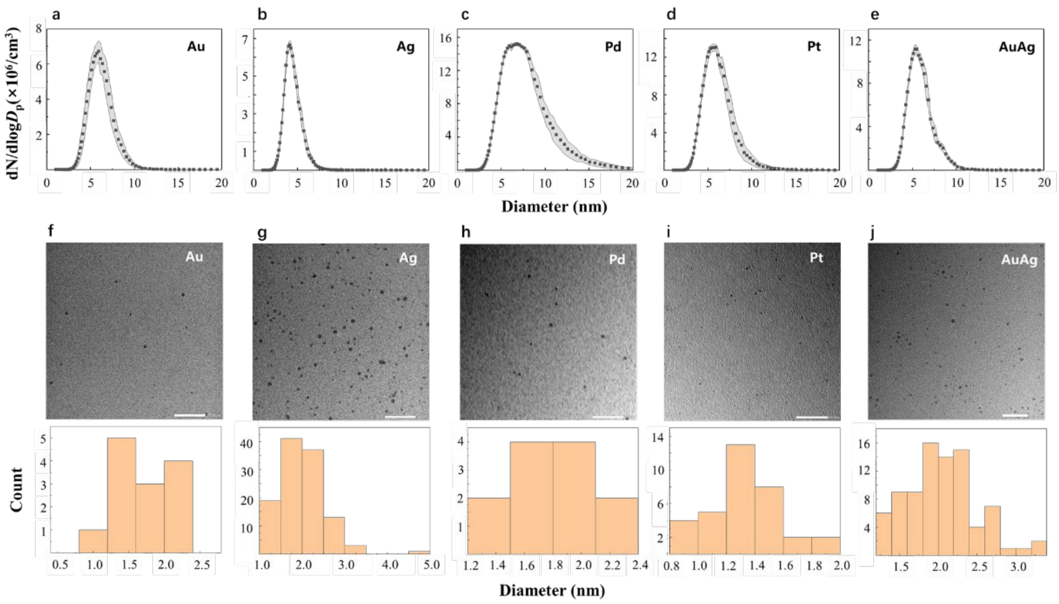

Supplementary Fig. 2: Size distribution of NPs measured by the NANO-SMPS and via analyzing their TEM (after size selection) images. a–e, Size distributions of Au (a), Ag (b), Pd (c), Pt (d) and Au–Ag (e) NPs repeated five times (the variations were presented in a shadow region), showing an averaged geometric mean diameter of 5.7 nm (Au), 4.2 nm (Ag), 6.7 nm (Pd), 5.6 nm (Pt) and 5.5 nm (Au–Ag) and an averaged geometric standard deviation of 1.22 (Au), 1.24 (Ag), 1.36 (Pd), 1.29 (Pt) and 1.26 (Au–Ag). **f–j,** TEM images and size statistics of Au (f), Ag (g), Pd (h), Pt (i) and Au–Ag (j) NPs after size-selection with a proper setting in Equation (1) for only allowing the printing of sub-3-nm particles. The scale bar is fixed to 20 nm. Source data are provided as a Source Data file.

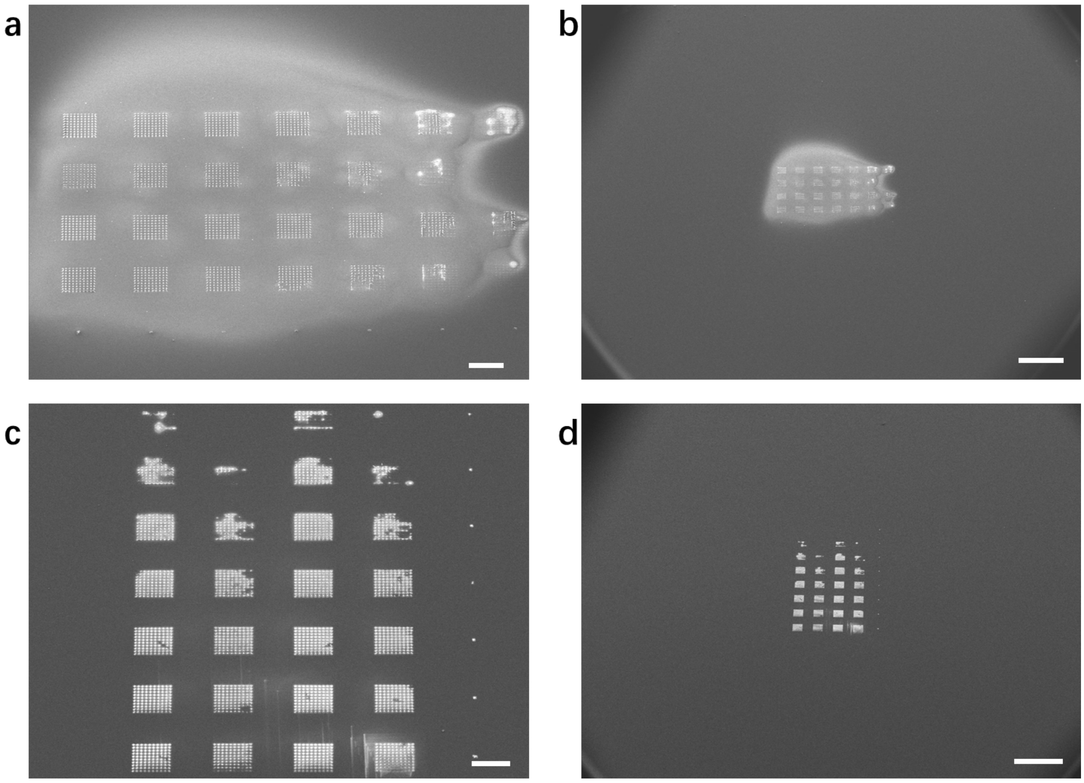

Supplementary Fig. 3: The effect of introducing a sheath flow used in tandem with electric fields. SEM images of PR surfaces after printing without (**a**, **b**) and with (**c**, **d**) the use of a clean gas. Scale bar for (**a**, **c**) is 100 μm and for (**b**, **d**) is 500 μm.

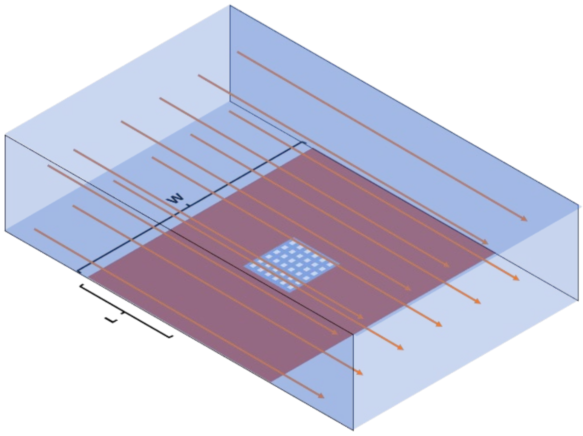

Supplementary Fig. 4: Schematic of printing zone. $S = W \times L$, represents a characteristic surface area that reflects a geometric volume of our printing zone. W represents the width of the print zone, and L represents the length between substrate edge and patterned area.

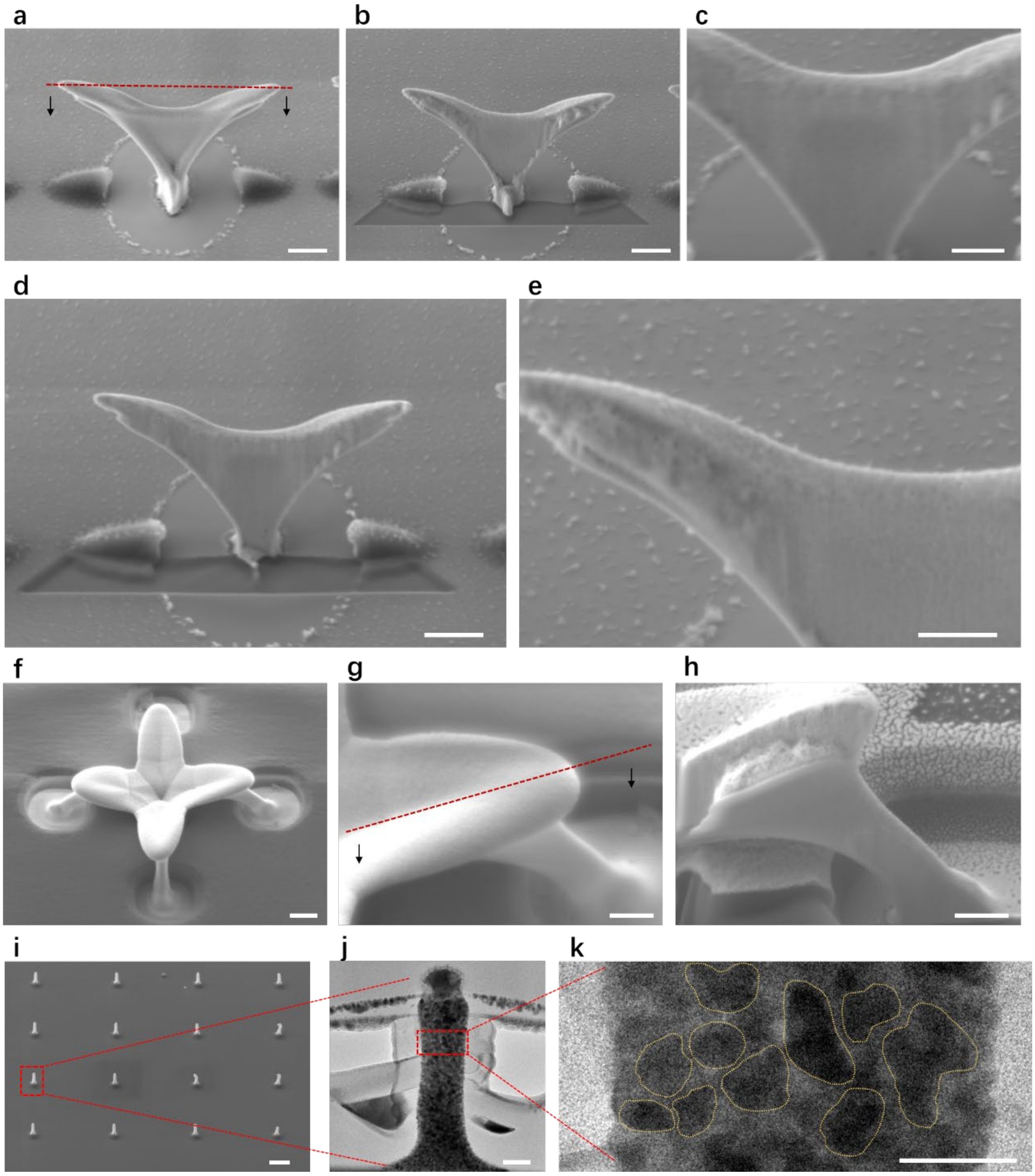

Supplementary Fig. 5: Interior nanostructures after FIB milling. a–h, SEM images of Au–Ag nanostructures before and after focused ion beam, showing that the interior structure is densely packed. Red dashed lines and arrow represent the initial place for FIB milling. **i–k**, SEM image (**i**) and TEM images (**j**, **k**) of the printed nanostructures before and after FIB milling, the dashed frames in **k** indicate that the particles have coalesced into larger grains. Scale bar for (**a**, **b, d, f, i**) is 1 μm, for (**c**, **e, g, h**) is 500 nm, for **j** is 100 nm and for **k** is 50 nm.

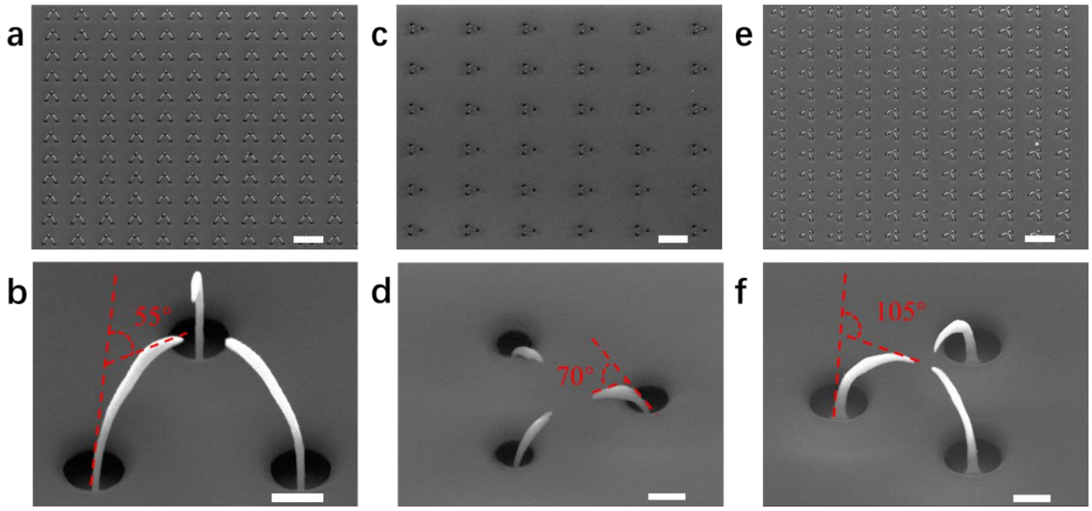

Supplementary Fig. 6: Arrayed nanowires with different bending angles and intergaps. **a**–**f**, SEM images of arrayed (**a**, **c**, **e**) and single (**b**, **d**, **f**) bent nanowires with different angles and materials. **a**, **b**, nanowires made of Au bent at an angle of 55°. **c**, **d**, nanowires made of Cu bent at an angle of 70°. **e**, **f**, nanowires made of Ag bent at an angle of 105°. Scale bar for (a, c, e) is 10 μm and for (b, d, f) is 1 μm.

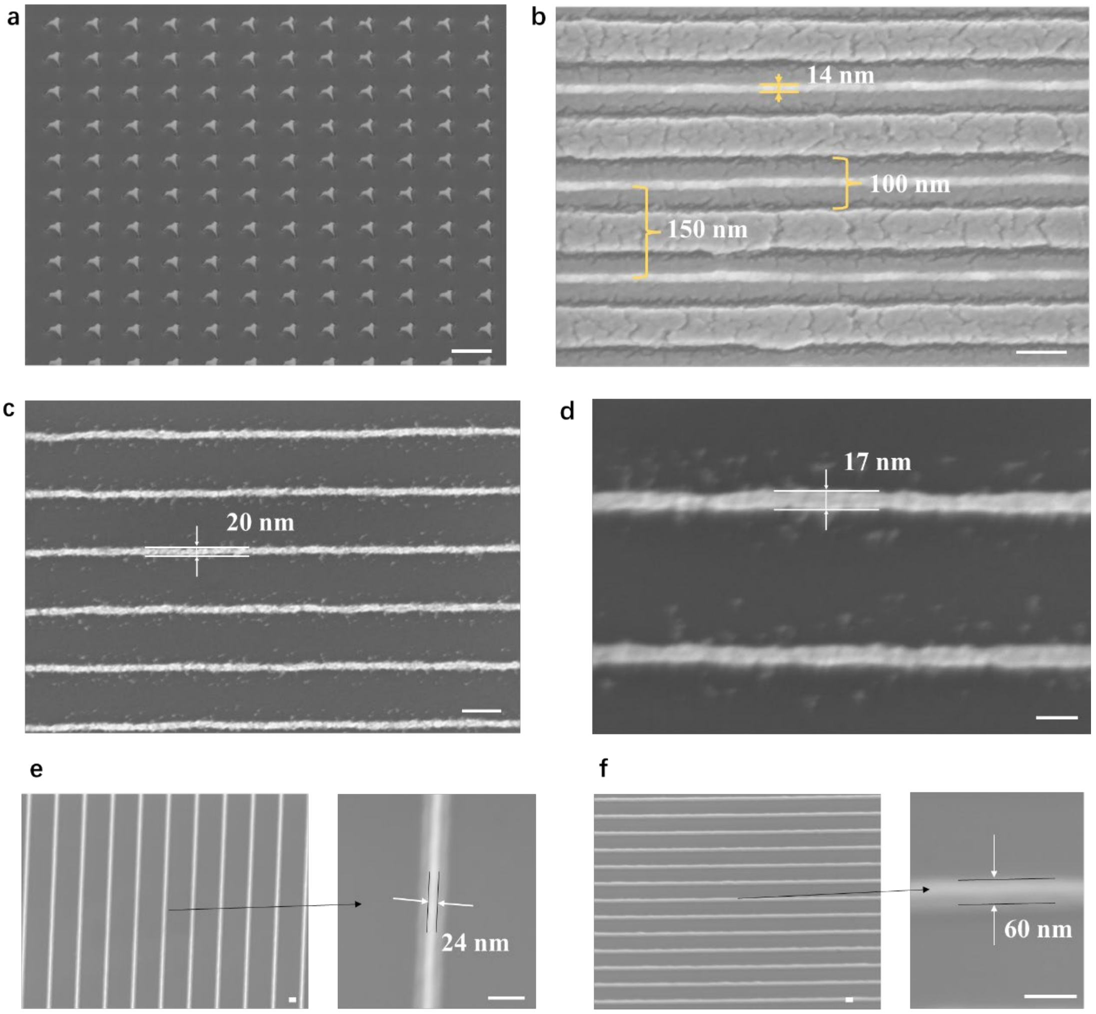

Supplementary Fig. 7: Nanoscale precision. **a**, SEM image of array nanostructures from Fig. 1j. **b**, An area of multiple line structures corresponding to the SEM image shown in Fig. 1k. The substrate for printing was patterned by an array of channels with a width of 150 nm, and the cracks formed due to sputtering Au layer on the PR surface. **c**, **d**, SEM images for showing sub-20-nm line width. **e**, **f**, Tilt views of the lines are actually true 3D structures, called nanowalls with an aspect ratio of approximately 3–5 (height: 60 nm, as marked in the right panel in **f** with a tilt angle of 53° for imaging, width: 24 nm, length: 10 000 nm). Scale bars: **a**, 1 μm; **b**, **c**, **e**, **f**, 100 nm; **d**, 40 nm.

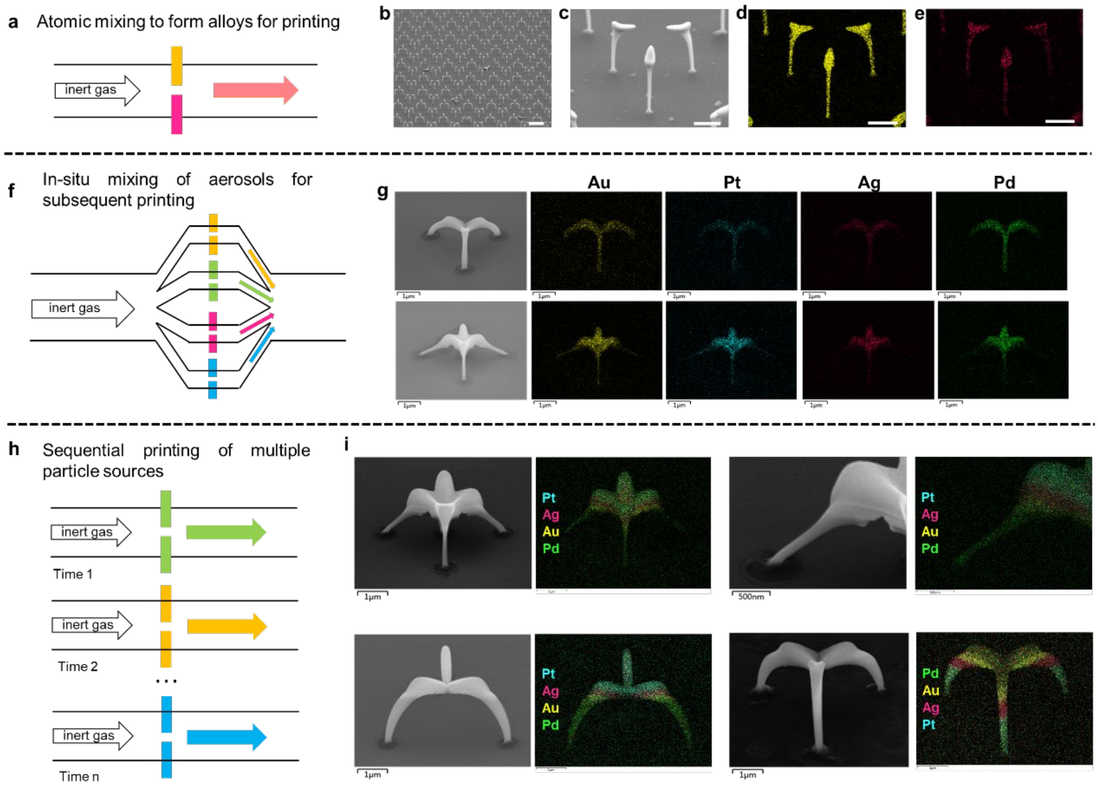

Supplementary Fig. 8: Multimaterials printing. Materials differences are marked in colors. **a**, Multimaterial printing: Atomic mixing. A pair of different electrodes was used in spark and generated alloy nanoparticles, which were subsequently printed. **b**, **c**, Mapping of the fields is flexibly controlled for printing periodic arrays of "ballet feet"–like structures made of an Au–Ag alloy, as confirmed by electron diffraction spectroscopy mapping for each element (**d**, **e**). Scale bar: **b**, 5 μm; **c**, **d**, **e**, 1 μm. **f**, Multimaterial printing: Nanoparticle–nanoparticle mixing. In–situ mixing of different aerosols from multiple particle sources run in parallel to form a mixed aerosol for subsequent printing. **h**, Multimaterial printing: segmented layers consisting of different materials of Pt, Ag, Au and Pd. Switching the particle source in chronological order to print multimaterials nanostructures in segmental forms. **g**, **i**, SEM images and the corresponding EDS mapping for the printed multimaterials nanostructures.

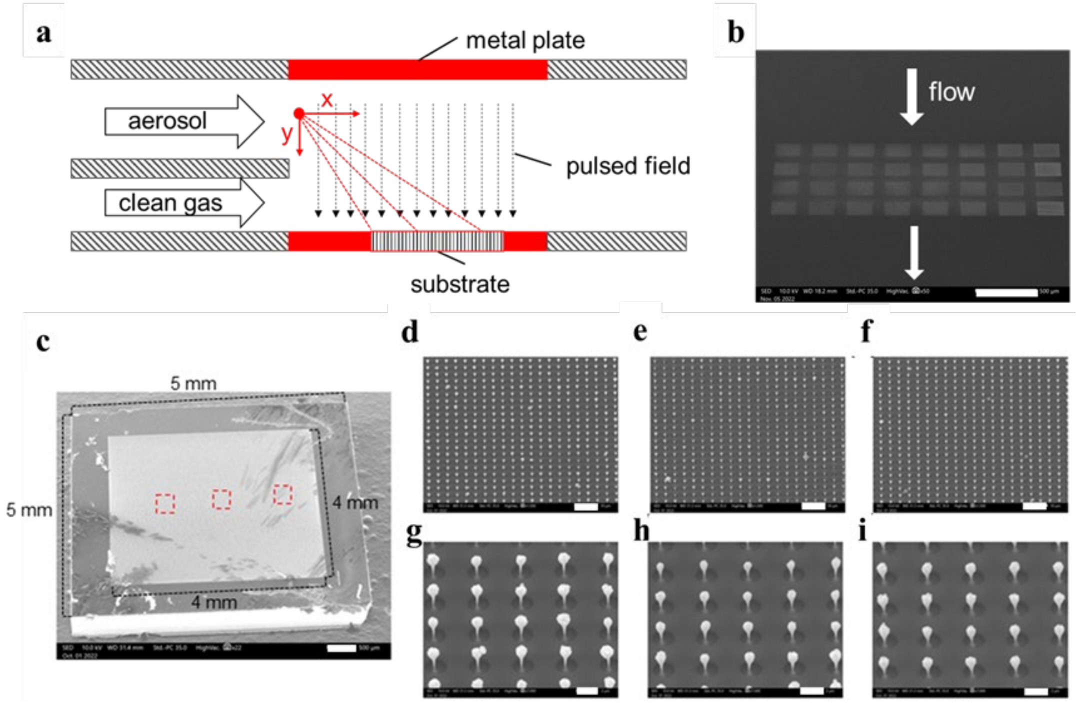

Supplementary Fig. 9: Printing metal 3D nanostructures over large areas. a, Schematics of the nanoprinter. Red area represents the printing area in which a pulsed electrical field was applied. Dashed lines refer to trajectories of NPs with different sizes. The arrows only indicated the direction. **b**, SEM image for showing the areas containing various arrays of the printed nanostructures. The entire area covered 2 × 1 mm^2. The image consisted 8 square areas in a row and 4 areas in a column and, in total, there were 32 areas distributed over the substrate. Each area had the printed nanostructure arrays. In printing, the gas flow was designed downward along the image, as marked by the arrows. **c**, SEM image of the printed nanostructures over an area of 4 × 4 mm^2 on a substrate with a dimension of 5 × 5 mm^2. Dashed squares marked the three representative locations of the printed nanostructures, whose SEM images are accordingly shown in **(d–f)**. **g–i**, Enlarged views of the SEM images for the nanostructures within the same area as shown in c–e. See Supplementary Discussion 2 for additional information for realizing large area printing. Scale bars: **b**, **c**, 500 μm; **d**, **e**, **f**, 10 μm; **g**, **h**, **i**. 2 μm.

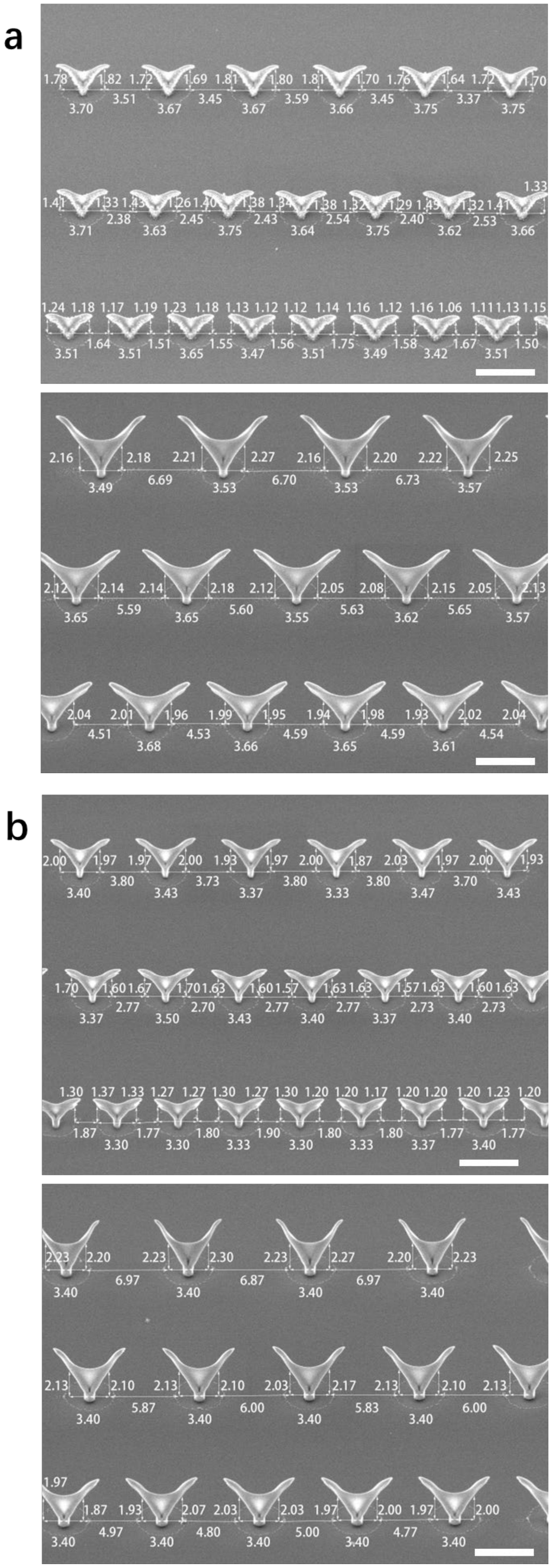
a
1.78 1.82 1.72 1.69 1.81 1.80 1.81 1.70 1.76 1.64 1.72 1.70
3.51 3.45 3.59 3.45 3.37
3.70 3.67 3.67 3.66 3.75 3.75
1.33
1.41 1.33 1.43 1.26 1.40 1.38 1.34 1.38 1.32 1.29 1.45 1.32 1.41
2.38 2.45 2.43 2.54 2.40 2.53
3.71 3.63 3.75 3.64 3.75 3.62 3.66
1.24 1.18 1.17 1.19 1.23 1.18 1.13 1.12 1.12 1.14 1.16 1.12 1.16 1.06 1.11 1.13 1.15
1.64 1.51 1.55 1.56 1.75 1.58 1.67 1.50
3.51 3.51 3.65 3.47 3.51 3.49 3.42 3.51
2.16 2.18 2.21 2.27 2.16 2.20 2.22 2.25
6.69 6.70 6.73
3.49 3.53 3.53 3.57
2.12 2.14 2.14 2.18 2.12 2.05 2.08 2.15 2.05 2.13
5.59 5.60 5.63 5.65
3.65 3.65 3.55 3.62 3.57
2.04 2.01 1.96 1.99 1.95 1.94 1.98 1.93 2.02 2.04
4.51 4.53 4.59 4.59 4.54
3.68 3.66 3.65 3.61
b
2.00 1.97 1.97 2.00 1.93 1.97 2.00 1.87 2.03 1.97 2.00 1.93
3.80 3.73 3.80 3.80 3.70
3.40 3.43 3.37 3.33 3.47 3.43
1.70 1.60 1.67 1.70 1.63 1.60 1.57 1.63 1.63 1.57 1.63 1.60 1.63
2.77 2.70 2.77 2.77 2.73 2.73
3.37 3.50 3.43 3.40 3.37 3.40
1.30 1.37 1.33 1.27 1.27 1.30 1.27 1.30 1.20 1.20 1.17 1.20 1.20 1.20 1.23 1.20
1.87 1.77 1.80 1.90 1.80 1.80 1.77 1.77
3.30 3.30 3.33 3.30 3.33 3.37 3.40
2.23 2.20 2.23 2.30 2.23 2.27 2.20 2.23
6.97 6.87 6.97
3.40 3.40 3.40 3.40
2.13 2.10 2.13 2.10 2.03 2.17 2.13 2.10 2.13
5.87 6.00 5.83 6.00
3.40 3.40 3.40 3.40
1.97
1.87 1.93 2.07 2.03 2.03 1.97 2.00 1.97 2.00
4.97 4.80 5.00 4.77
3.40 3.40 3.40 3.40 3.40

c

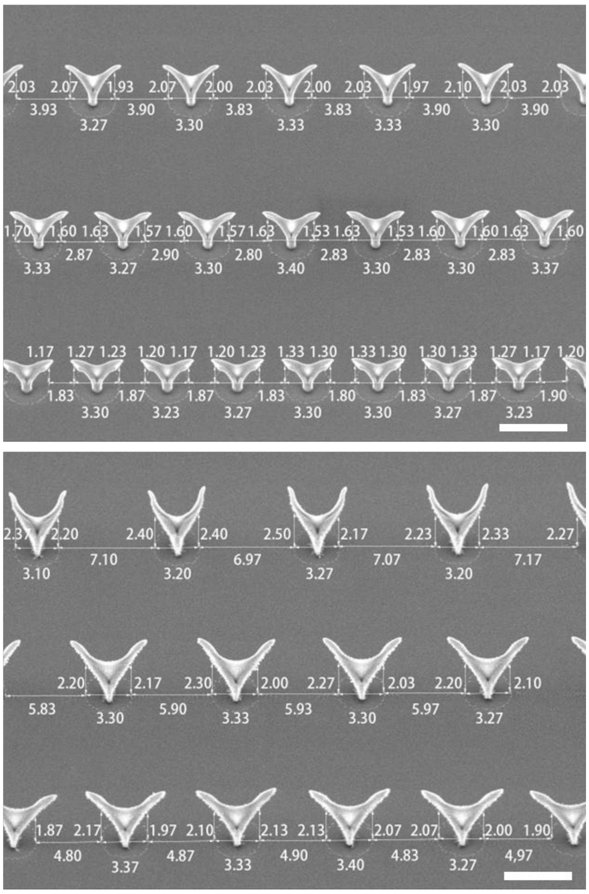
2.03 2.07 1.93 2.07 2.00 2.03 2.00 2.03 1.97 2.10 2.03 2.03
3.93 3.90 3.83 3.83 3.90 3.90
3.27 3.30 3.33 3.33 3.30
1.70 1.60 1.63 1.57 1.60 1.57 1.63 1.53 1.63 1.53 1.60 1.60 1.63 1.60
2.87 2.90 2.80 2.83 2.83 2.83
3.33 3.27 3.30 3.40 3.30 3.30 3.37
1.17 1.27 1.23 1.20 1.17 1.20 1.23 1.33 1.30 1.33 1.30 1.30 1.33 1.27 1.17 1.20
1.83 1.87 1.87 1.83 1.80 1.83 1.87 1.90
3.30 3.23 3.27 3.30 3.30 3.27 3.23
2.37 2.20 2.40 2.40 2.50 2.17 2.23 2.33 2.27
7.10 6.97 7.07 7.17
3.10 3.20 3.27 3.20
2.20 2.17 2.30 2.00 2.27 2.03 2.20 2.10
5.83 5.90 5.93 5.97
3.30 3.33 3.30 3.27
1.87 2.17 1.97 2.10 2.13 2.13 2.07 2.07 2.00 1.90
4.80 4.87 4.90 4.83 4,97
3.37 3.33 3.40 3.27

d

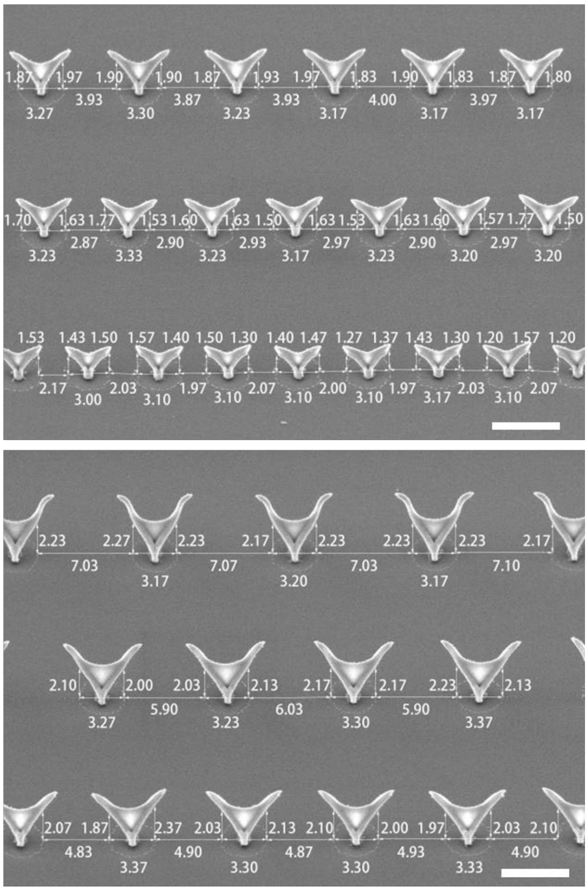
1.87 1.97 1.90 1.90 1.87 1.93 1.97 1.83 1.90 1.83 1.87 1.80
3.93 3.87 3.93 4.00 3.97
3.27 3.30 3.23 3.17 3.17 3.17
1.70 1.63 1.77 1.53 1.60 1.63 1.50 1.63 1.53 1.63 1.60 1.57 1.77 1.50
2.87 2.90 2.93 2.97 2.90 2.97
3.23 3.33 3.23 3.17 3.23 3.20 3.20
1.53 1.43 1.50 1.57 1.40 1.50 1.30 1.40 1.47 1.27 1.37 1.43 1.30 1.20 1.57 1.20
2.17 2.03 1.97 2.07 2.00 1.97 2.03 2.07
3.00 3.10 3.10 3.10 3.10 3.17 3.10
2.23 2.27 2.23 2.17 2.23 2.23 2.23 2.17
7.03 7.07 7.03 7.10
3.17 3.20 3.17
2.10 2.00 2.03 2.13 2.17 2.17 2.23 2.13
5.90 6.03 5.90
3.27 3.23 3.30 3.37
2.07 1.87 2.37 2.03 2.13 2.10 2.00 1.97 2.03 2.10
4.83 4.90 4.87 4.93 4.90
3.37 3.30 3.30 3.33

e

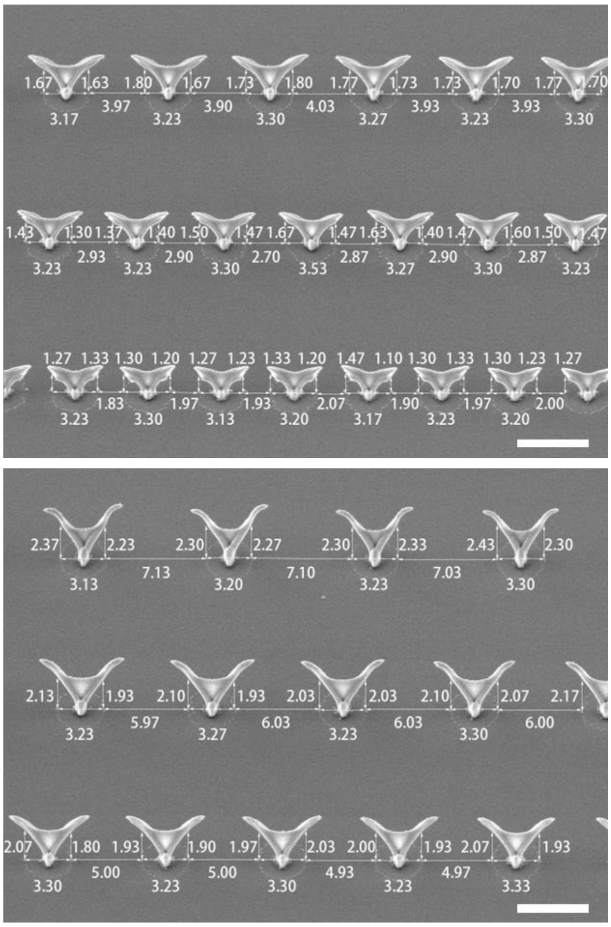

Supplementary Fig. 10: Marked sizes for evaluating the cushion field. a–e, numerically marked cushion heights at an applied potential of 400 V (a), 500 V (b), 600 V (c), 700 V (d) and 800 V (e) without calibration with the tilt angle. The units for the numerical values marked here are all microns. The scale bar is fixed to 5 μm. Source data are provided as a Source Data file and the values therein were directly obtained from those shown in the images and thus not calibrated with the tilt angle.

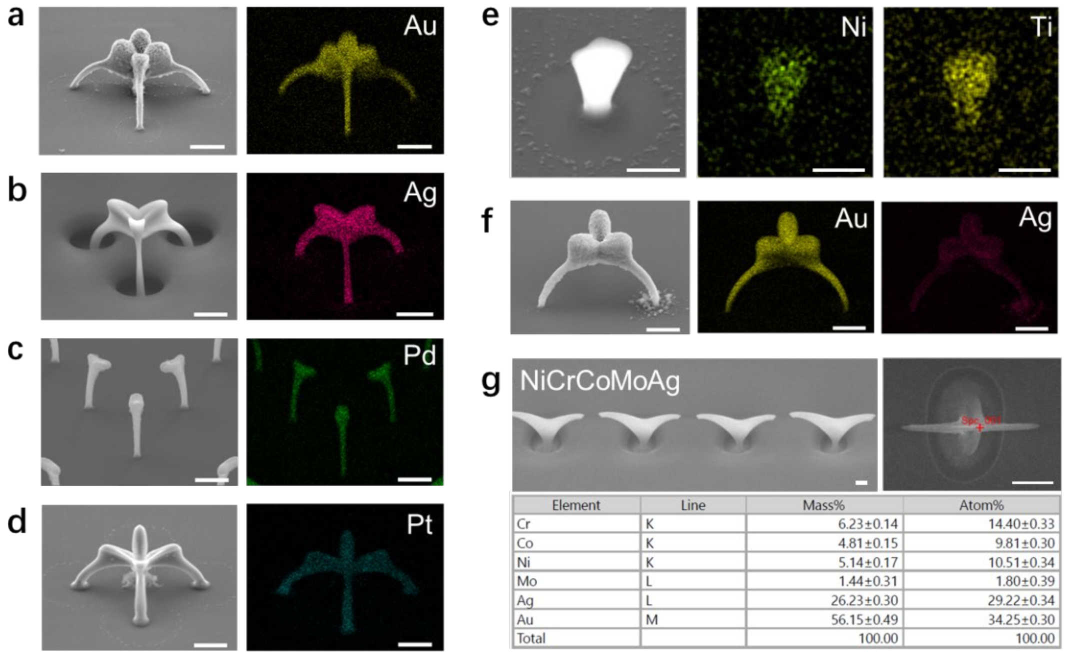

Element	Line	Mass%	Atom%
Cr	K	6.23±0.14	14.40±0.33
Co	K	4.81±0.15	9.81±0.30
Ni	K	5.14±0.17	10.51±0.34
Mo	L	1.44±0.31	1.80±0.39
Ag	L	26.23±0.30	29.22±0.34
Au	M	56.15±0.49	34.25±0.30
Total		100.00	100.00

Supplementary Fig. 11: 3D nanoprinting of different metals. a–f, SEM images and EDS mapping analysis of Au (**a**), Ag (**b**), Pd (**c**), Pt (**d**), Ni–Ti (**e**) and Au–Ag (**f**) nanostructures. **g**, SEM images and EDS point analysis of Ni–Cr–Co–Mo–Ag nanostructure, where Au signal was from substrate because a Au layer was coated for enhancing the adhesion of the nanostructures to the substrate. The scale bar is fixed to 1 μm.

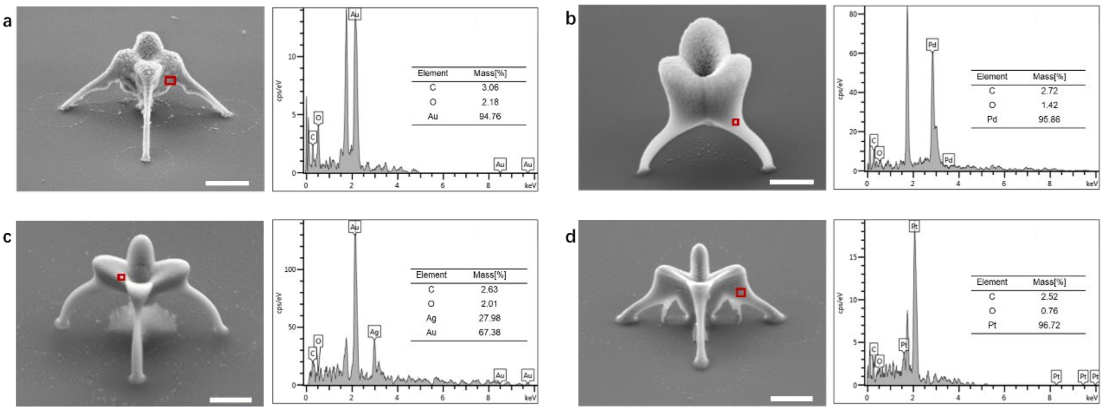

Supplementary Fig. 12: EDS analysis of metal 3D nanostructures. a–d, EDS analysis of Au (**a**), Pd (**b**), Au–Ag (**c**) and Pt (**d**) nanostructures. Data show EDS area (marked by a red box) spectra for each material. The nanostructures consist of almost pure Au (more than 94 wt%), Pd (more than 95 wt%), Au–Ag (> 95 wt%) and Pt (more than 96 wt%) because O and C are always present owing to the exposure of the sample to the environment. The scale bar is fixed to 1 μm.

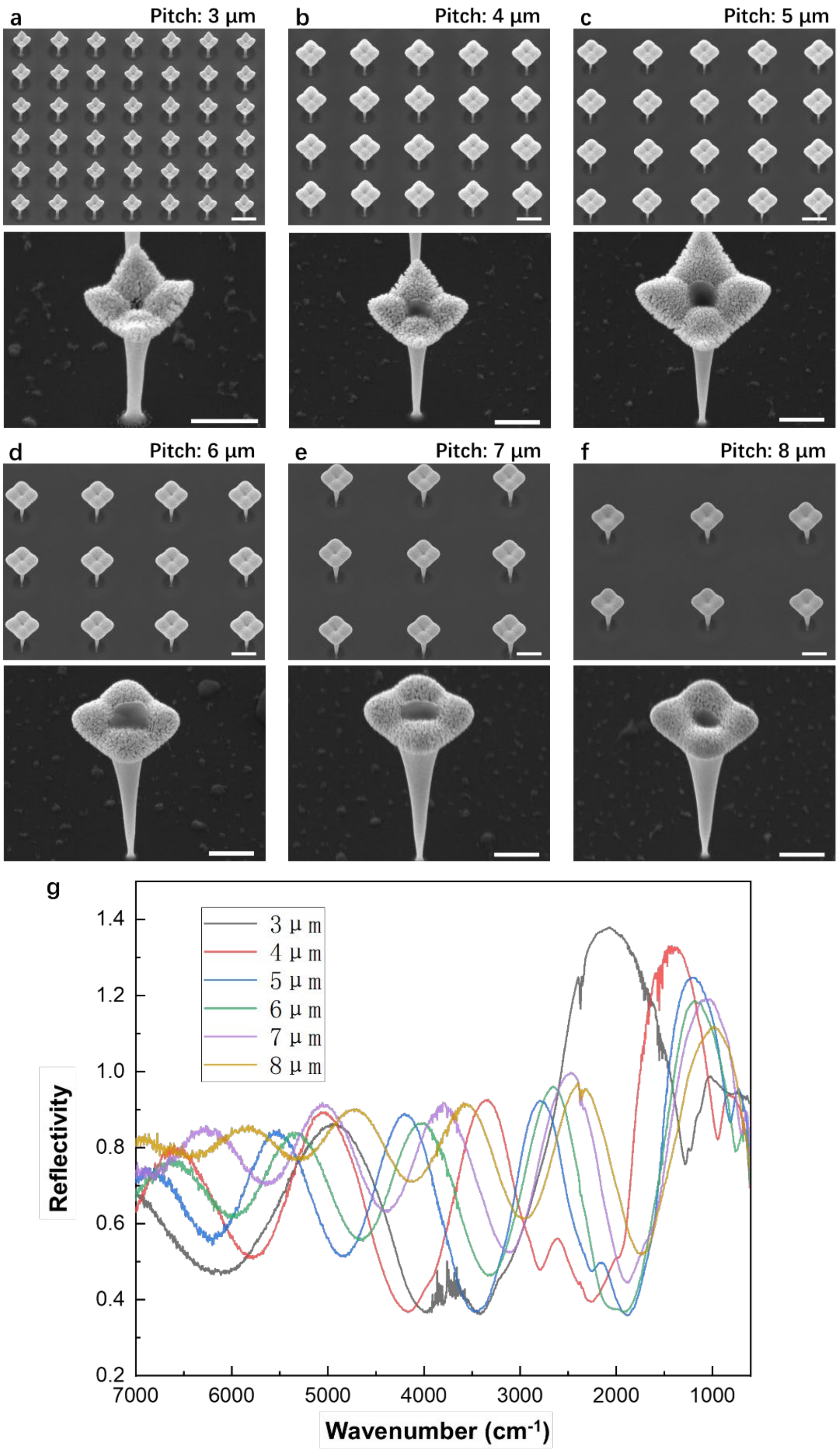

Supplementary Fig. 13: IR measurements of the metal nanostructures. a–f, SEM images for nanostructure arrays with a pitch of 3 μm (**a**), 4 μm (**b**), 5 μm (**c**), 6 μm (**d**), 7 μm (**e**) and 8 μm (**f**). **g**, the infrared reflectance results of the printed nanostructure arrays. Source data are provided as a Source Data file.

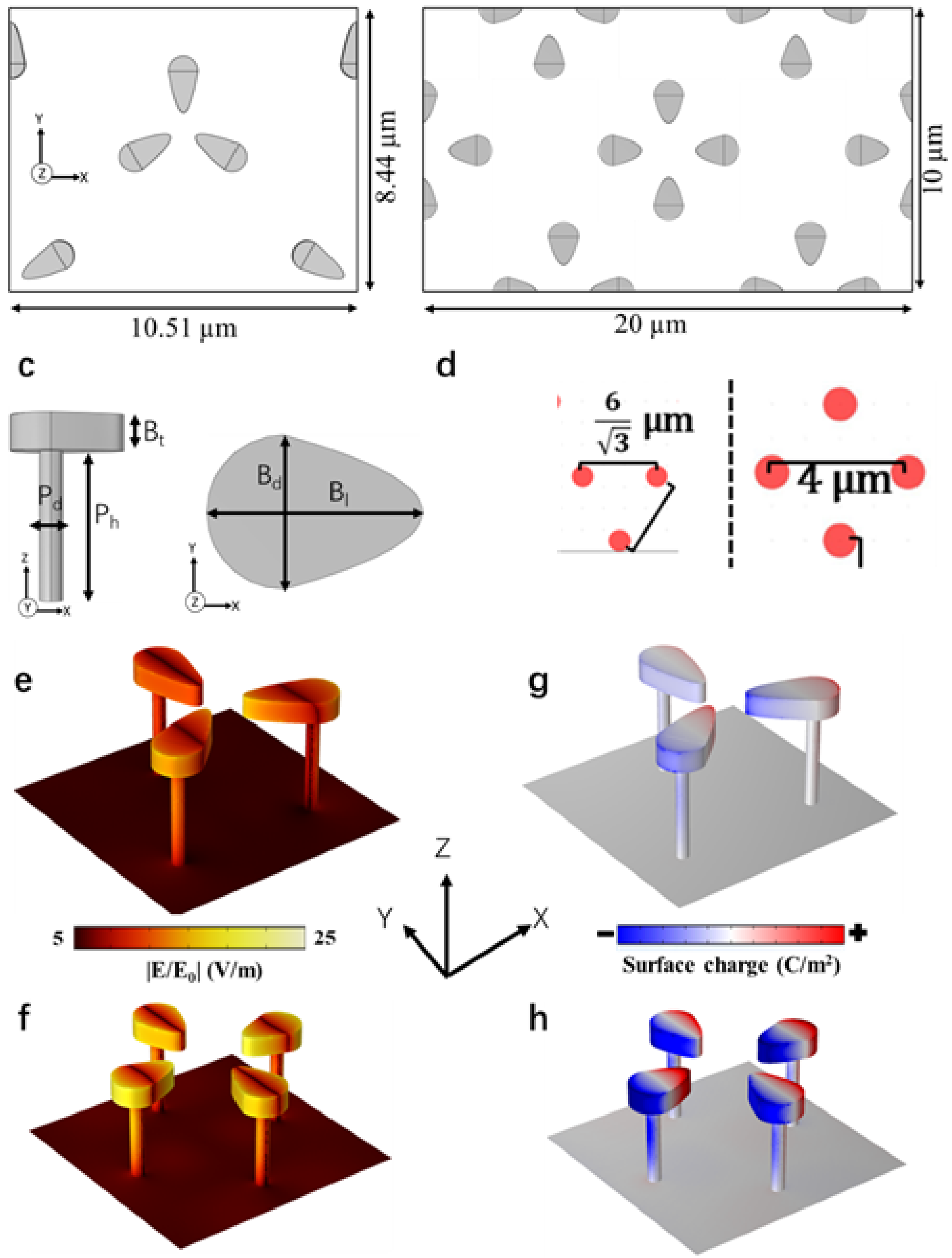

Supplementary Fig. 14: Simulated results for light interactions with the metal 3D nanostructures. Top view of (XY direction) unit cell model of (**a**) 3-bead and (**b**) 4-bead structure with periodic boundary condition information. (**c**) Geometry details: Bead's diameter, length, and thickness are termed B_d, B_l, and B_t, respectively. Pillar diameter and height are given as P_d and P_h, respectively. For 3-bead structure: B_d = 900 nm, B_l = 1700 nm, B_t = 400 nm, P_d = 230 nm, and P_h = 2 µm. For 4-bead structure: B_d = 920 nm, B_l = 1290 nm, B_t = 400 nm, P_d = 250 nm, and P_h = 1.7 µm. (**d**) Spacing information for 3- and 4-bead structures. All the above parameters are for only Pd-involved structures. The coated Au thickness on Pd is 7 nm. The spacing is fixed from pillar center to pillar center. 3D electric field profiles (**e**, **f**) and corresponding surface charge density distribution mappings (**g**, **h**) taken at their respective resonance wavelength positions for 3-bead and 4-bead structure.

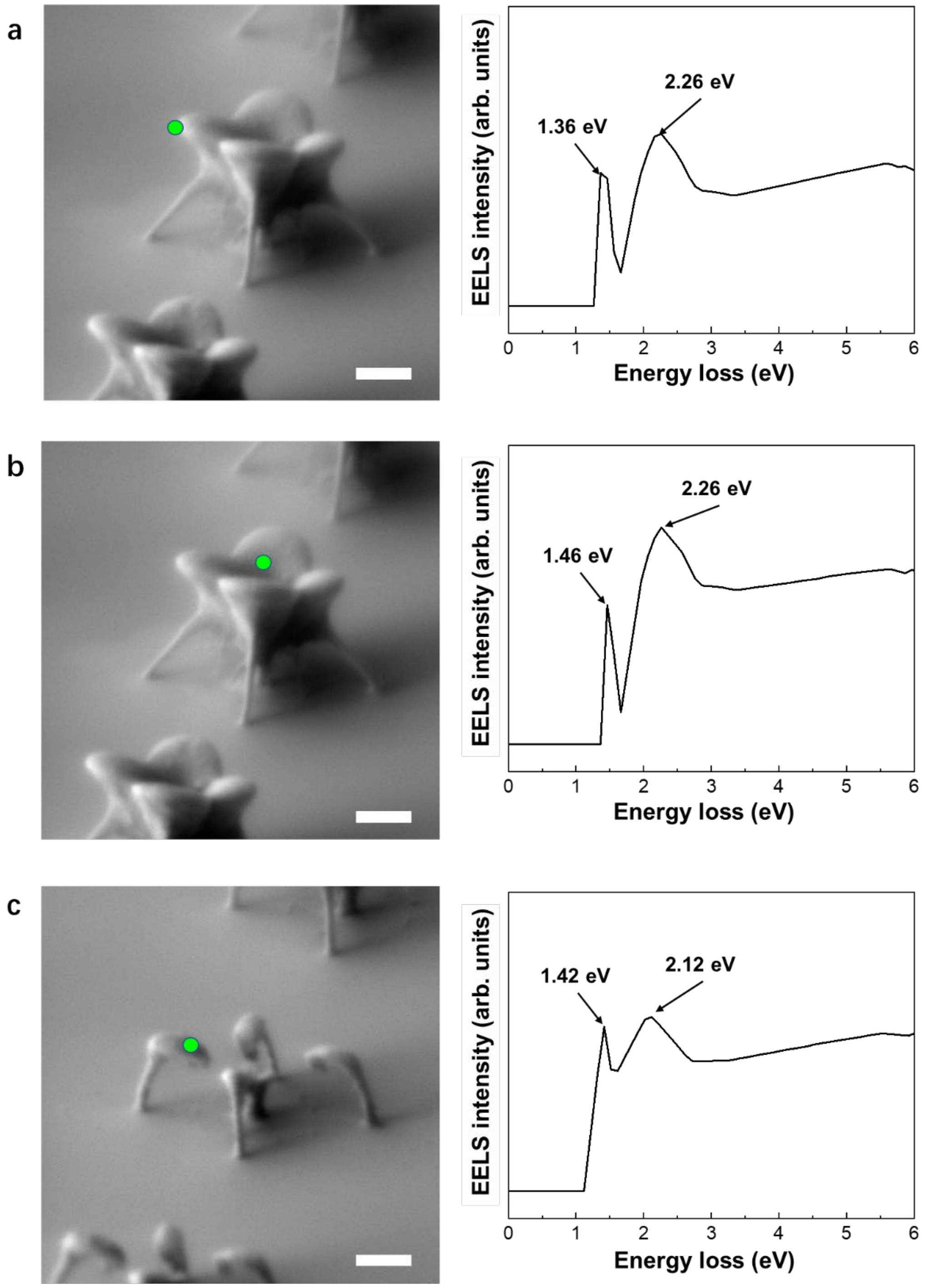

Supplementary Fig. 15: REELS measurements for the Pd nanostructures. a–**c**, SEM images of Pd nanostructures and their REELS data. The signal collection position has been marked with a green dot in the images. The scale bar is fixed to 1 μm. Source data are provided as a Source Data file.

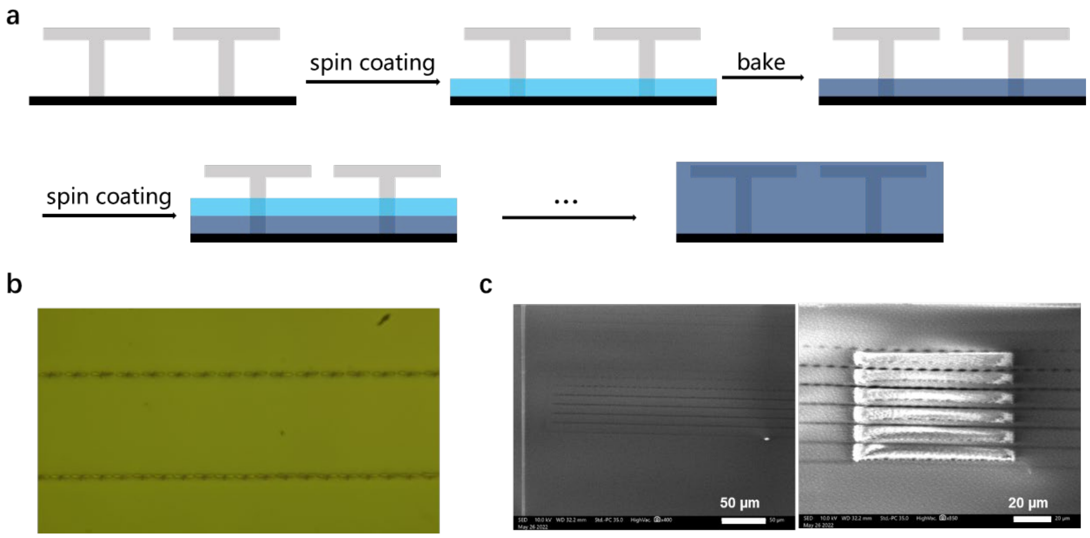

Supplementary Fig. 16: Protocols for surface protection. a, Process flow for making structural protection. The printed nanostructures were buried by the photoresist to avoid subsequent oxidation and/or mechanical damage. **b**, Optical microscope image and SEM images (**c**) of the structural array after photoresist coverage.

Supplementary Tables

Supplementary Table 1 Tilt angles used in SEM imaging

**Figure**	Fig. 1j	Fig. 1d, e, i	Fig. 1h, l–o; Fig. 3; Fig. 4	Fig. 2
**Title angle (°)**	30	45	53	60

Supplementary Table 2 Pattern design

**Primary unit: Number of holes**				
**3**	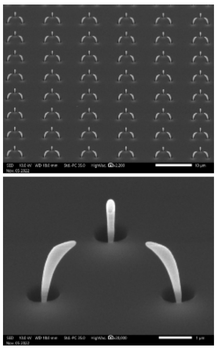	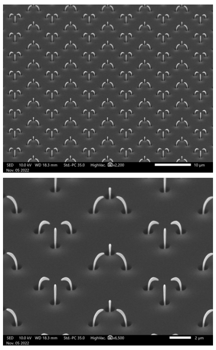	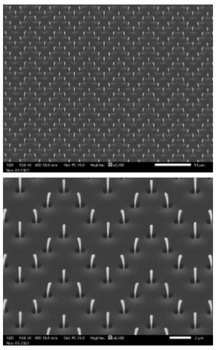	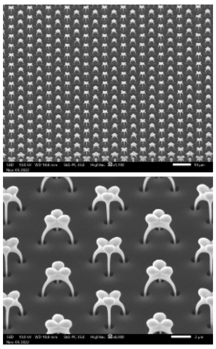
**4**	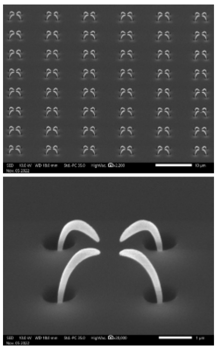	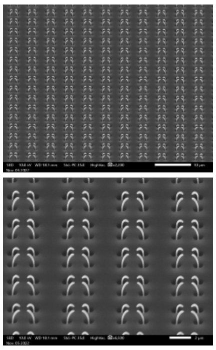	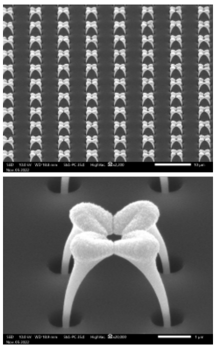	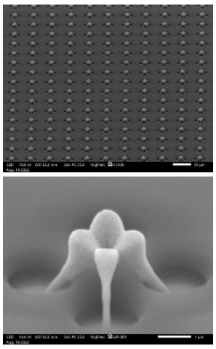
**5**	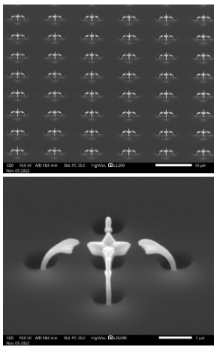	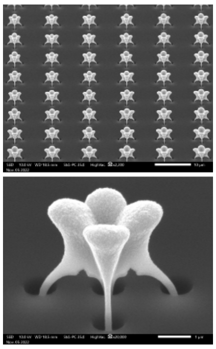	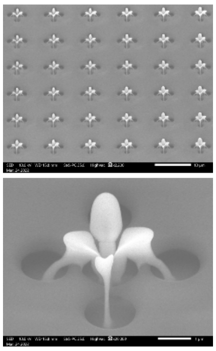	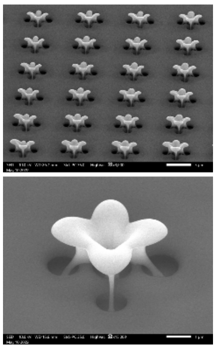
**6**	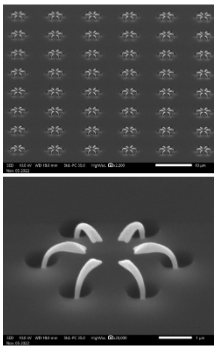	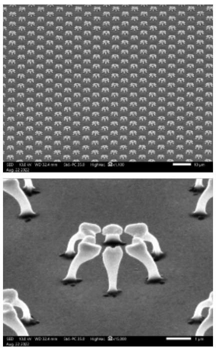	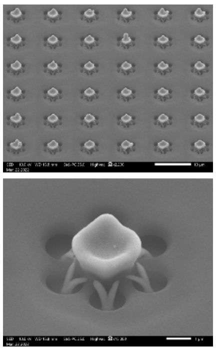	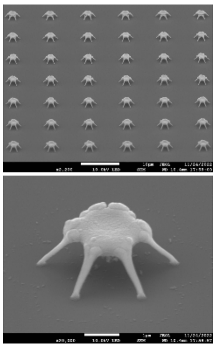

Supplementary Table 3 Experimental parameters for printing multimaterials nanostructures

			**Pd**	**Au**	**Ag**	**Pt**
**Multi-material printing: NP–NP mixing**	Particle source	Breakdown voltage (kV)	1.4	1.6	1.6	1.5
		Frequency (Hz)	170	490	700	140
		Capacitance (nF)	1	1	2	1
	Printing conditions	Clean gas flow rate (lpm)	1.2			
		Aerosol flow rate (lpm)	2			
		Field strength (kV cm^{-1})	0.625			
		Printing time (min)	240			
**Multimaterials printing: segmental layers consisting of different materials (in the order of Pd– Au– Ag– Pt arranged upward)**	Particle source	Breakdown voltage (kV)	1.5	1.7	1.8	1.4
		Frequency (Hz)	390	340	600	430
		Capacitance (nF)	1	1	2	1
	Printing conditions	Clean gas flow rate (lpm)	1.2			
		Aerosol flow rate (lpm)	2			
		Field strength (kV cm^{-1})	0.625			
		Printing time (min)	50	60	240	50
**Multimaterials printing: segmental layers consisting of different materials (in the order of Pt– Ag– Au– Pd**	Particle source	Breakdown voltage (kV)	1.6	1.7	1.9	1.4
		Frequency (Hz)	360	340	590	420
		Capacitance (nF)	1	1	2	1
	Printing conditions	Clean gas flow rate	1.2			

**arranged upward)**	(lpm)				
	Aerosol flow rate (lpm)	2			
	Field strength (kV cm^{-1})	0.625			
	Printing time (min)	50	90	180	50

Supplementary Table 4 Flow rates and potentials used in printing

Voltage	- 400V	- 500 V	- 600 V	-700 V	-800 V
**Aerosol flow (lpm)**	2.4	3	3.6	4.2	4.8
**Sheath flow (lpm)**	0.8	1	1.2	1.4	1.6

https://doi.org/10.1038/s41467-023-40577-3

Supplementary Table 5 Pattern designs for the substrates used for printing the structures.

The hole diameter in the pattern was fixed to 800 nm, which can be treated as a scale bar for gaining more information about the geometrical designs of the patterned substrates.

	Au	Ag	Pd	Pt	Au–Ag
a	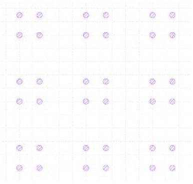	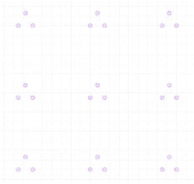	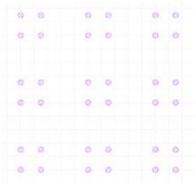	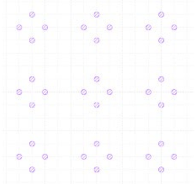	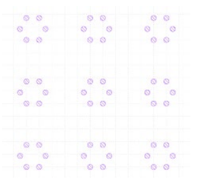
b	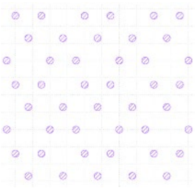	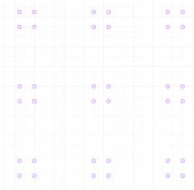	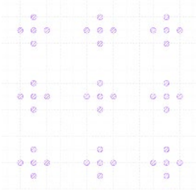	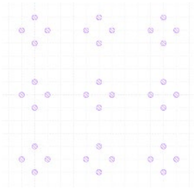	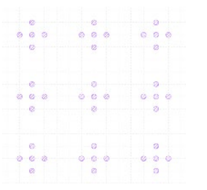
c	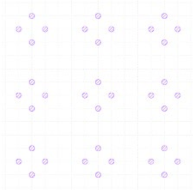	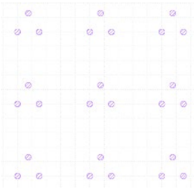	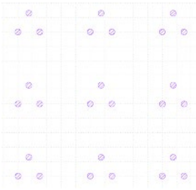	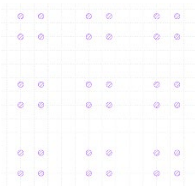	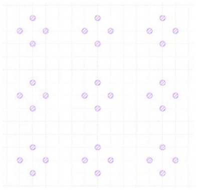
d	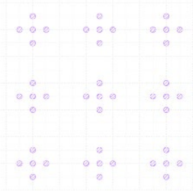	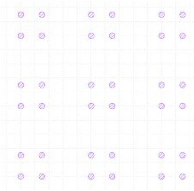	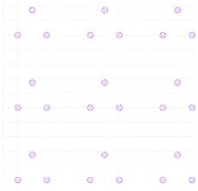	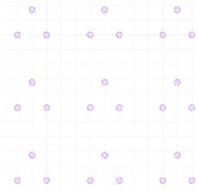	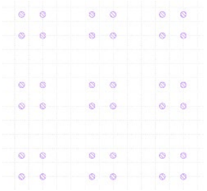
e	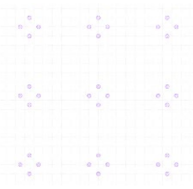	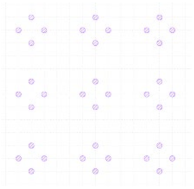	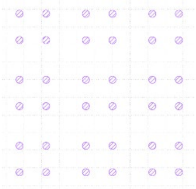	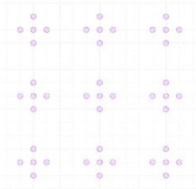	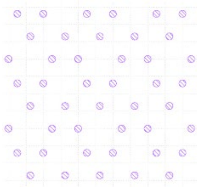
f	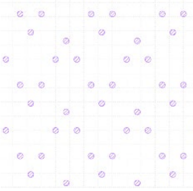	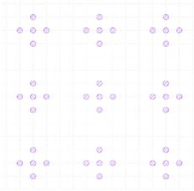	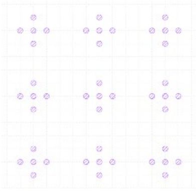	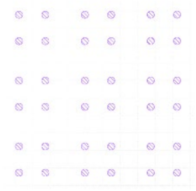	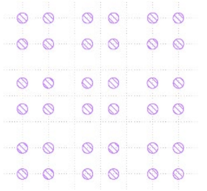
g	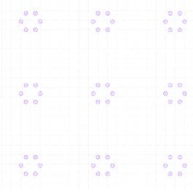	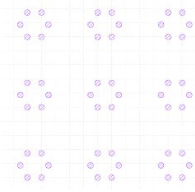	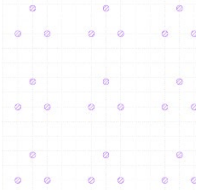	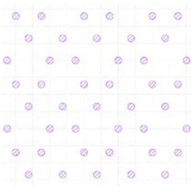	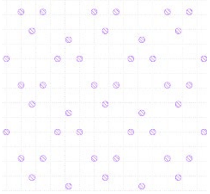

h

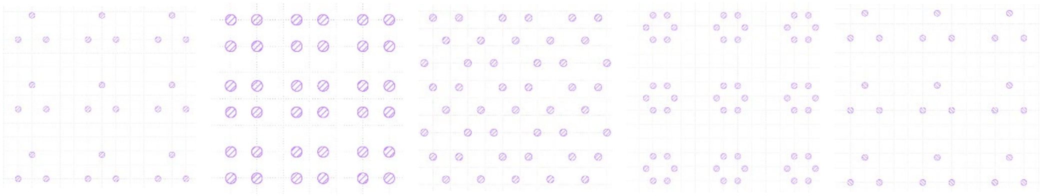

Supplementary Discussion

Supplementary Discussion 1. Particle size-selection with coupled flow and electric fields

According to the definition of electrical mobility:

$$Z_p = \frac{neC}{3\pi\eta D_p};$$

where C is the Cunningham correction factor:

$$C = 1 + \frac{2\lambda}{D_p}\left[1.165 + 0.483 \cdot \exp\left(-\frac{0.997D_p}{2\lambda}\right)\right];$$

Charged particles are subjected to electric force that is vertical to the flow field, and the termination velocity of the particles in the electric field is:

$$V_{\mathrm{TE}} = Z_{\mathrm{p}}E;$$

Neglecting the relaxation time for the nanoparticles to reach the termination velocity, the charged particles released from the top of the clean gas flow move at the same speed as the flow field in lateral direction, and are dragged by the electric field force in the vertical direction to move at the termination velocity. For the charged particles reaching the distance L:

$$\frac{L}{u_s} = \frac{h_s}{V_{\mathrm{TE}}}$$

Put into the above formula to get:

$$\frac{D_{\mathrm{p}}}{C} = \frac{neEL}{3\pi\eta h_{\mathrm{s}}u_{\mathrm{s}}} = \frac{ne}{3\pi\eta}Lw\,\frac{E}{Q_s} = \left(S\frac{e}{3\pi\eta}\right)\frac{E}{Q_s}$$

Where L and W accordingly represent the distance from the edge of the substrate to the printing area and the width of the substrate (Supplementary Fig. 4).

To maintain a smooth transition between the two layers of the flow, the aerosol flow was adjusted accordingly when performing size selection (detailed parameters are listed in Table S3).

The MATLAB code used to plot the relationship between the flow and electric field for exerting the size selection of the nanoparticles is as follows:

```
η=        ; % gas dynamic viscosity
λ=        ; % gas mean free path
e=        ; % elementary charge
α=1.165;
β=0.483;
γ=0.997; % Parameters in the Cunningham Correction Factor[1]
f=@(R,D)(1+(2.* λ./(D*1e-9)).*( α + β.*exp(-γ.*(D*1e-9)./(2.*
λ)))).*(e.*R*6e7*(20*1e-6)./(3.*pi.* η))-(D*1e-9); % D: Particle diameter,
R=E/Q
fimplicit(f,[0 200 0 10]);
```

Supplementary Discussion 2. Methodology of printing uniform nanostructures over large areas

Inspired by an aerosol sampler able to uniformly deposit aerosol onto a large flat surface[2], we applied pulsed electrical field during printing process to realize the large area printing for one pass only. Two key parameters for this are the location of the substrate and the duty cycle of the pulse field (controlled by a switching circuit that is home-made). Below we describe how to determine these parameters.

Based on the particle motion in the coupled fields, the location of substrate is determined by:

$$\begin{cases} y = Z_{\min} \cdot E \cdot t \\ x_0 \geqslant v_{gas} \cdot t \end{cases}$$

Where Z_{min} is the electrical mobility of the largest particle, v_{gas} is the velocity of aerosol gas flow and E is the electrical field intensity.

The duty cycle is determined by:

$$\begin{cases} t_1 = \dfrac{y}{Z_p \cdot E} \\ t_2 = \dfrac{L}{v_{gas}} \end{cases}$$

Where L is the length of the printing area along the direction of the aerosol flow, t_1 represents a printing duration and t_2 is the time for the aerosol flow to transport downstream with the absence of the electric field.

Supplementary References

1. Kim, J. H., Mulholland, G. W., Kukuck, S. R. & Pui, D. Y. H. Slip Correction Measurements of Certified PSL Nanoparticles Using a Nanometer Differential Mobility Analyzer (Nano-DMA) for Knudsen Number From 0.5 to 83. *J. Res. Natl. Inst. Stand. Technol.* **110**, 31–54 (2005).
2. Liu, B. Y. H., Whitby, K. T. & Yu, H. H. S. Electrostatic Aerosol Sampler for Light and Electron Microscopy. *Rev. Sci. Instrum.* **38**, 100–102 (1967).
